# Peptide Fractions Extracted from the Hemolymph of *Hermetia illucens* Inhibit Growth and Motility and Enhance the Effects of Traditional Chemotherapeutics in Human Colorectal Cancer Cells

**DOI:** 10.3390/ijms26051891

**Published:** 2025-02-22

**Authors:** Donatella Lucchetti, Roberta Rinaldi, Giulia Artemi, Rosanna Salvia, Federica De Stefano, Carmen Scieuzo, Patrizia Falabella, Alessandro Sgambato

**Affiliations:** 1 Department of Translational Medicine and Surgery, Università Cattolica del Sacro Cuore, 00168 Rome, Italy; donatella.lucchetti@unicatt.it (D.L.); giulia.artemi@unicatt.it (G.A.); 2 Multiplex Spatial Profiling Facility, Fondazione Policlinico Universitario ‘Agostino Gemelli’ IRCCS, 00136 Rome, Italy; 3 Department of Basic and Applied Sciences, University of Basilicata, Via dell’Ateneo Lucano 10, 85100 Potenza, Italy; roberta.rinaldi@unibas.it (R.R.); r.salvia@unibas.it (R.S.); federica.destefano@unibas.it (F.D.S.); carmen.scieuzo@unibas.it (C.S.); 4 Centro di Riferimento Oncologico della Basilicata IRCCS (IRCCS-CROB), 85028 Rionero in Vulture, Italy; 5 Spinoff XFlies S.R.L., University of Basilicata, Via Dell’Ateneo Lucano 10, 85100 Potenza, Italy

**Keywords:** anticancer peptides, in vitro anticancer activity, insects

## Abstract

Cancer is a leading cause of death worldwide, characterized by uncontrolled cell growth and multiple mutations. Chemotherapy is often associated with harmful side effects, and cancer cells may become resistant through various mechanisms. New approaches, which are able to address both the toxicity and resistance issues of chemotherapy, are of primary importance in cancer research. Antimicrobial peptides (AMPs), naturally occurring molecules in the innate immune system of all living organisms, have a wide spectrum of cytotoxic activities against cancer cells and could be a promising alternative to actual chemotherapeutics. Here, we tested peptide fractions, rich in AMPs, extracted from the hemolymph of the larvae of the insect *Hermetia illucens* on the HT29 and HCT116 human colorectal cancer cells, observing cell growth inhibition by cell accumulation in the G2/M phase and increased apoptosis. Furthermore, the peptide extract induced a significant cytoskeleton reorganization, resulting in reduced motility. These effects were more evident with the peptide fractions obtained from the *Escherichia coli*-infected larvae. The peptide fractions also enhanced the effects of traditional chemotherapeutics. Overall, the results obtained suggest the presence of biologically active molecules in the hemolymph of *H. illucens* larvae, confirming that insect-derived peptides are a promising research area in oncology.

## 1. Introduction

Cancer is one of the leading causes of death worldwide and is characterized by the uncontrolled growth of cells that accumulate multiple mutations, as well as epigenetic alterations, over the years [1,2]. These changes may be caused by physical, chemical, and/or biological agents and/or inherited genetic factors [3,4].

Despite advances in oncology, conventional chemotherapy remains the primary treatment for many types of cancer. However, its effectiveness is often compromised by severe side effects, as it indiscriminately targets both cancerous and healthy cells [5]. In addition, cancer cells can develop resistance to chemotherapeutic drugs through various mechanisms, including the overexpression of detoxifying enzymes and/or drug carriers that are able to reduce the intracellular drug concentration, alterations in the concentration of drug targets, the increased ability to repair DNA damage or tolerate stress conditions, and defects in the apoptotic pathways [5,6].

To overcome these limitations, researchers are actively investigating novel therapeutic strategies based on the use of drugs and/or a combination of drugs that are able to overcome resistance by effectively blocking multiple pathways and on the use of molecules with specific and targeted activity on cancer cells, with no limiting doses, thus avoiding any long-term and off-target toxic effects on healthy cells while maximizing toxic effects on cancer cells [6]. Among these approaches, the interest in the study of peptides with antitumor activity has exponentially increased in the last few decades, and numerous studies have shown that antimicrobial peptides (AMPs), naturally occurring peptides that are a component of the innate immune system in all living organisms, defending them against several pathogenic agents, including bacteria, viruses, and fungi, can display a wide spectrum of cytotoxic activity against cancer cells. Such peptides are referred to as ACPs, anticancer peptides [7,8,9], and have proven effective against cancer cells, encouraging the exploration of their potential as therapeutic agents alone and/or in combination with existing chemotherapeutics or other targeted therapies [8,9,10,11,12].

AMPs are small, amphipathic molecules typically characterized by a sequence of amino acids with usually 5 to 50 residues, high hydrophobicity, and a net charge value ranging from −5 (anionic antimicrobial peptides—AAPs) to +10 (cationic antimicrobial peptides—CAPs—which are rich in residues of lysine and arginine) [13,14]. Although anionic peptides with anticancer activity have been found, CAPs are the most abundant AMPs, acting by selectively and electrostatically interacting and disrupting cancer cell membranes (membranolytic actions) and/or by entering the cells and interacting with intracellular components (non-membranolytic actions). Cancer cells, similar to bacteria, have a net negative charge, as they express more anionic molecules, like phosphatidylserine and O-glycosylated mucins, on the cytoplasmic membrane’s outer sheet than normal cells [15,16,17,18]. Furthermore, compared to healthy cells, cancer cells usually have higher membrane fluidity [19,20], resulting in easier membrane destabilization by membrane-bound CAPs [19,20]. The surface of cancer cells often has a greater presence of microvilli than that of healthy cells, which increases the contact surface and makes it easier for CAPs to interact with the outside of cancer cells [21]. In addition, the mitochondria of eukaryotic cells have a highly negative transmembrane potential, whereby, after entering the cell, CAPs with non-membranolytic activity can damage the integrity of negatively charged mitochondria membranes [22], causing more mitochondrial proteins to be released into the cytosolic space, resulting in the activation of apoptotic pathways [12,23].

AMPs are produced by all living organisms, but insects, which constitute about 55% of the total biodiversity on Earth, are among the richest and most innovative sources of AMPs [24]. In particular, as reported in many other papers on the insect *Hermetia illucens* L. (Diptera: Stratiomyidae), also known as the Black Soldier Fly (BSF), represents an extraordinarily rich source of AMPs, which is probably due to its ability to live in hostile environments, its ability to feed and bioconvert decaying substrates, and the fact that it is rich in microbial colonies [25,26,27,28,29].

A previously reported overview of *H. illucens* AMPs identified in different transcriptomes, that were analyzed *in silico* in order to characterize them for chemo-physical properties and putative biological activities, found that some of them were predicted to be endowed with anticancer activity (ACPs) [26]. Since AMPs are primarily released into the hemolymph, the insect circulatory fluid, in response to microbial infections, their extraction from infected larvae represents a promising strategy to identify biologically active peptides with potential anticancer effects [30,31].

In this study, we investigated the potential anticancer activity of Peptide Fractions (PFs) extracted from the hemolymph of *H. illucens* larvae, either uninfected or infected with two different bacteria. This approach was based on previous predictions of putative ACPs in *H. illucens* and the well-documented preferential release of AMPs into the hemolymph in response to microbial infections. The results, obtained using in vitro models of colorectal cancer cells (CRCs), suggest the presence of biologically active peptides in the hemolymph, thus confirming that the study of insect-derived peptides with antitumor activity is a promising area of research in oncology, with the potentiality to allow for the identification of new effective anticancer agents.

## 2. Results

### 2.1. Inhibitory Effects of H. Illucens-Derived Peptide Fractions on CRC Cells’ Viability

**Figure 1:**
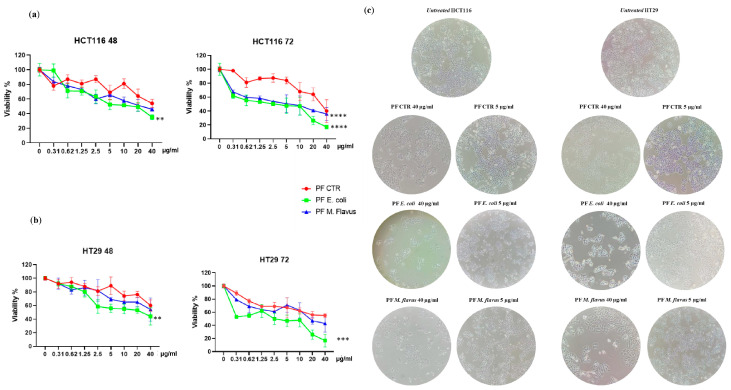
*H. illucens*-derived peptide fractions affect the viability and morphology of human CRC cells. Cell viability assay on (**a**) HCT116 and (**b**) HT29 cells. Cytotoxicity of the peptide fractions derived from uninfected and infected larvae with *M. flavus* and *E. coli* of *H. illucens* larvae was assessed on HT29 and HCT116 cells treated with different concentrations of samples (from 0.31 to 40 µg/mL) for 48 h and 72 h. The percentage of viable cells was calculated as the ratio of treated cells to control cells. Data are presented as means ± Standard Error (SE) of three technical replicates of three independent assays. Statistical significance between groups was evaluated by one-way analysis of variance (ANOVA) followed by Dunnett’s post hoc test (GraphPad Prism 5 software). The difference was considered statistically significant at ** *p* < 0.01, *** *p* < 0.001, and **** *p* < 0.0001. (**c**) Morphological cell analysis. Microscopic analysis showing changes in the morphology of HT29 and HCT116 cells induced by the IC_50_ of peptide fractions after 48 h of treatment. Magnification: 10×; scale bars: 80 µm.

To evaluate the effects of Peptide Fractions (PFs) extracted from the hemolymph of *Hermetia illucens* larvae on colorectal cancer cells (CRC), we performed a dose–response experiment by treating HT29 and HCT116 cells with several dilutions (40 µg/mL, 20 µg/mL, 10 µg/mL, 5 µg/mL, 2.5 µg/mL 1.25 µg/mL, 0.62 µg/mL, and 0.31 µg/mL) of the peptide fractions obtained from larvae infected with *Escherichia coli* or *Micrococcus flavus* and from non-infected larvae. Cell viability was assessed after 48 h and 72 h using the MTT test. A dose- and time-dependent inhibition of cell growth in both cell lines was observed, and this effect was stronger with the peptide fractions from infected larvae compared to the peptide fractions from uninfected larvae. Moreover, peptide fractions from larvae infected with *E. coli* displayed a higher ability to inhibit cancer cell growth compared to the ones from *M. flavus*-infected larvae on both cell lines (Figure 1a,b).

A microscopic analysis of the cell cultures confirmed the peptide fractions’ inhibitory effect and evidenced different degrees of cellular deformation and distention, with these cells losing their normal shape and appearing slimmer than the control, untreated cells (Figure 1c).

### 2.2. Peptide Fractions Induced G2/M Phase Arrest and Apoptosis in Human CRC Cells

**Figure 2:**
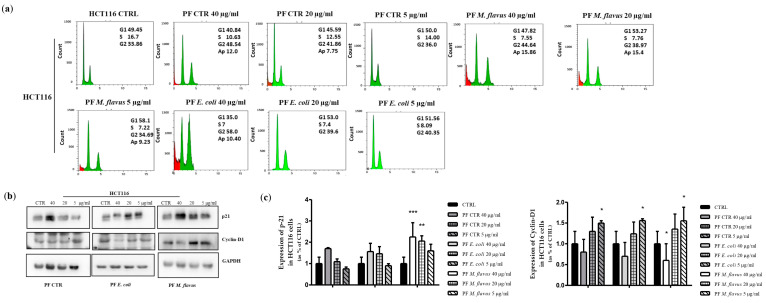
*H. illucens*-derived peptide fractions affect cell cycle distribution in HCT116 human CRC cells. (**a**) Flow cytometry analysis. Cell cycle analysis demonstrated a dose-dependent accumulation of cells in the G2/M phase of the cell cycle (in green) in all treatment groups after 48 h of treatment compared to control cells. This effect was stronger in the cultures treated with peptide fractions from infected larvae than the uninfected counterpart and was more evident with the peptide fractions obtained from larvae infected with *E. coli*. The apoptotic peak is indicated in red. (**b**) Western blotting analysis of cell cycle-related proteins. Protein expression was evaluated by immunoblotting after a 48 h treatment with the indicated peptide fractions at three different concentrations. A densitometric analysis (**c**) was performed by the ImageJ-win64 software and is reported by histograms. Data are presented as means ± the Standard Error (SE) of three technical replicates of three independent assays. Statistical significance between groups was evaluated by one-way analysis of variance (ANOVA), followed by Dunnett’s post hoc test (GraphPad Prism 5 software). The difference was considered statistically significant at * *p* < 0.05, ** *p* < 0.01, and *** *p* < 0.001.

**Figure 3:**
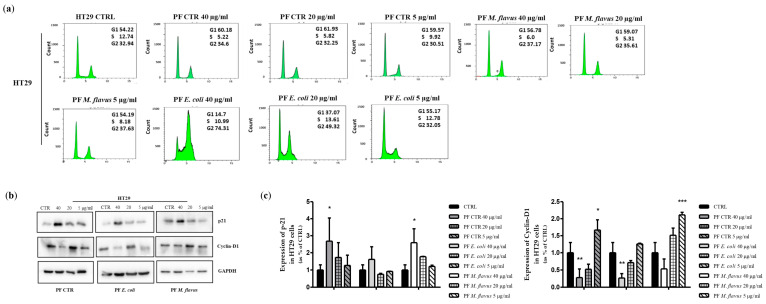
*H. illucens*-derived peptide fractions affect cell cycle distribution in HT29 human CRC cells. (**a**) Flow cytometry analysis. Cell cycle analysis demonstrated a dose-dependent accumulation of cells in the G2/M phase of the cell cycle (in green) in all treatment groups after 48 h of treatment compared to control cells. This effect was stronger in the cultures treated with peptide fractions from infected larvae than the uninfected counterpart and was more evident with the peptide fractions obtained from larvae infected with *E. coli*. (**b**) Western blotting analysis of cell cycle-related proteins. Protein expression was evaluated by immunoblotting after a 48 h treatment with the indicated peptide fractions at three different concentrations. A densitometric analysis (**c**) was performed by the ImageJ-win64 software and is reported by histograms. Data are presented as means ± the Standard Error (SE) of three technical replicates of three independent assays. Statistical significance between groups was evaluated by one-way analysis of variance (ANOVA), followed by Dunnett’s post hoc test (GraphPad Prism 5 software). The difference was considered statistically significant at * *p* < 0.05, ** *p* < 0.01, and *** *p* < 0.001.

In order to understand the mechanisms mediating the observed inhibition of cell growth, the distribution of cells within the phases of the cell cycle was analyzed using flow cytometry in both the HCT116 (Figure 2a) and HT29 (Figure 3a) cell cultures treated with the peptide fractions. A cell cycle analysis demonstrated that the peptide fractions induced cell cycle arrest. In fact, after 48 h hours of treatment, a dose-dependent accumulation of cells in the G2/M phase of the cell cycle was observed in both cell lines and with all the treatments compared to the control, untreated cells. Similar to growth inhibition, this effect was stronger in the cultures treated with peptide fractions isolated from infected larvae than the uninfected counterpart and was more evident with the peptide fractions obtained from larvae infected with *E. coli* (Figure 2a and Figure 3a).

In the cultures treated with the peptide fractions obtained from larvae infected with *E. coli*, the percentage of cells in the G2/M phase of the cell cycle reached 74% and 58% in the HT29 and the HCT116 cells, respectively, compared to the about 33% in the control, untreated cells. A slight effect (37% and 44%, respectively, in the HT29 and HCT116 cells) was observed with the peptide fractions obtained from larvae infected with *M. flavus* (Figure 2a and Figure 3a).

To investigate the molecular mechanisms underlying the obtained results, we investigated the effects of peptide fractions on the expression of proteins known to be involved in the regulation of the cell cycle. An increase in the expression levels of the p21 protein (CDK inhibitor) and a decreased expression of cyclin-D1 was detected in both cell lines compared to the untreated cells (Figure 2b,c and Figure 3b,c). This effect was the highest with the peptide fractions obtained from larvae infected with *E. coli*. A flow cytometry analysis also revealed the appearance of a sub-G1 peak, indicative of apoptotic cells, in the HCT116 cells treated with the peptide fractions obtained from larvae infected with *E. coli* or *M. flavus*, which reached a value of about 10% and 16% at 48 h, respectively (Figure 2a). A sub-G1 peak was not evident in the cultures of treated HT29 cells (Figure 3a).

**Figure 4:**
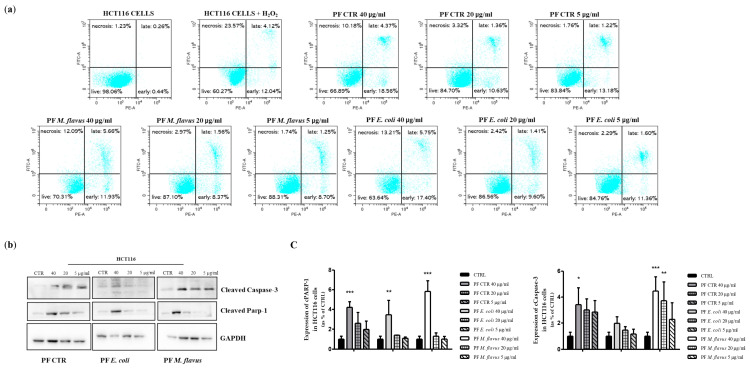
*H. illucens*-derived peptide fractions induced apoptosis in HCT116 human CRC cells. (**a**) Quantitative analysis of apoptosis. A quantitative evaluation of apoptosis after 48 h of treatment of HCT116 cells with the indicated increasing concentrations of peptide fractions showed a dose-dependent increase in the number of apoptotic cells. (**b**) Western blotting analysis of cell cycle-related proteins. Protein expression was evaluated by immunoblotting after 48 h of treatment with the indicated peptide fractions at three different concentrations. A densitometric analysis (**c**) was performed by the ImageJ-win64 software and is reported by histograms. Data are presented as means ± the Standard Error (SE) of three replicates. Statistical significance between groups was evaluated by one-way analysis of variance (ANOVA), followed by Dunnett’s post hoc test (GraphPad Prism 5 software). The difference was considered statistically significant at * *p* < 0.05, ** *p* < 0.01, and *** *p* < 0.001.

To further evaluate the potential pro-apoptotic effect of the hemolymph-derived peptide fractions, we analyzed the occurrence of apoptosis in the peptide fraction-treated cultures of both cell lines using the PI/AnnexinV double-staining assay. The results obtained confirm the occurrence of apoptosis in the treated HCT116, but not HT29, cells (Figure 4 and Supplementary Figure S1). Indeed, the percentage of apoptotic cells after 48 h of treatment reached the early apoptosis values of 18%, 17%, and 12% in the HCT116 cultures treated with peptide fractions that were obtained from larvae infected with *E. coli* and *M. flavus* and from uninfected larvae, respectively (Figure 4a). Moreover, we detected a percentage of late apoptosis values of 6%, 5%, and 4%, respectively (Figure 4a). Apoptosis was confirmed by the dose-dependent increase in cleaved caspase-3 and cleaved Parp1 in the HCT116 cells treated with an increasing amount of peptide fractions compared to untreated cells (Figure 4b). No induction of apoptosis was observed in the HT29 cells (Figure 3a and Supplementary Figure S1).

### 2.3. Peptide Fractions Increased Motility and Affected Cytoskeleton Organization in Human CRC Cells

**Figure 5:**
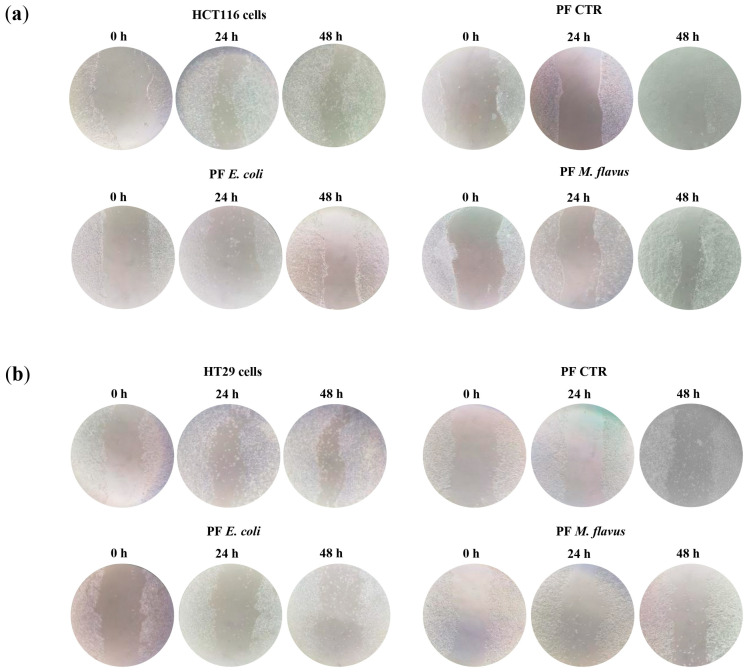
*H. illucens*-derived peptide fractions reduced the motility of human CRC cells. Scratch test analysis. A total of 2 × 10^5^ cells per well of (**a**) HCT116 and (**b**) HT29 cell lines were plated in 24-well plates and were allowed to reach confluence. A scratch wound was made using a pipette tip. Media were replaced, and cultures were exposed to peptide fractions and were photographed at different time points. The samples delayed the closure of the wound in a dose-dependent manner. Magnification: 10×; scale bars: 80 µm.

**Figure 6:**
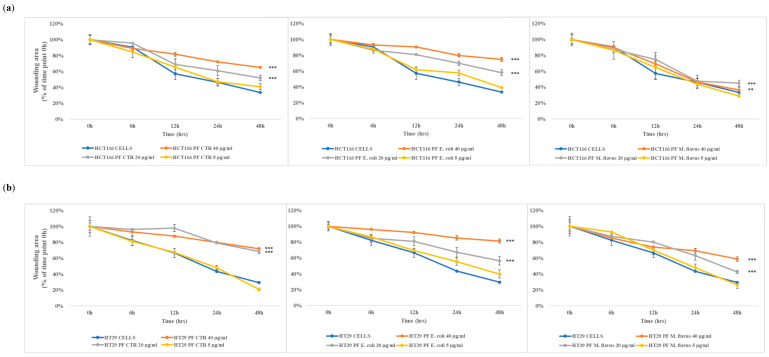
Wound area analysis of HCT116 and HT29 cells over time. The wound area for both (**a**) HCT116 and (**b**) HT29 was calculated by the ImageJ software at different time points (time: 0 h, 6 h, 12 h, 24 h, and 48 h). Statistical significance between groups was evaluated by two-way analysis of variance (ANOVA), followed by Bonferroni’s post hoc test (GraphPad Prism 5 software). The difference was considered statistically significant at ** *p* < 0.01, and *** *p* < 0.001.

To further investigate the effects of peptide fractions on the phenotype of human CRC lines, we used the scratch-wound migration assay to analyze two-dimensional cell migration in the control and treated HCT116 and HT29 cell cultures (Figure 5 and Figure 6).

The results show that peptide fractions slowed down wound closures compared to the untreated cells in a dose-dependent manner, with wound healing time increasing as peptide fraction concentrations rise, and this effect was the highest with the peptide fractions obtained from larvae infected with *E. coli*.

**Figure 7:**
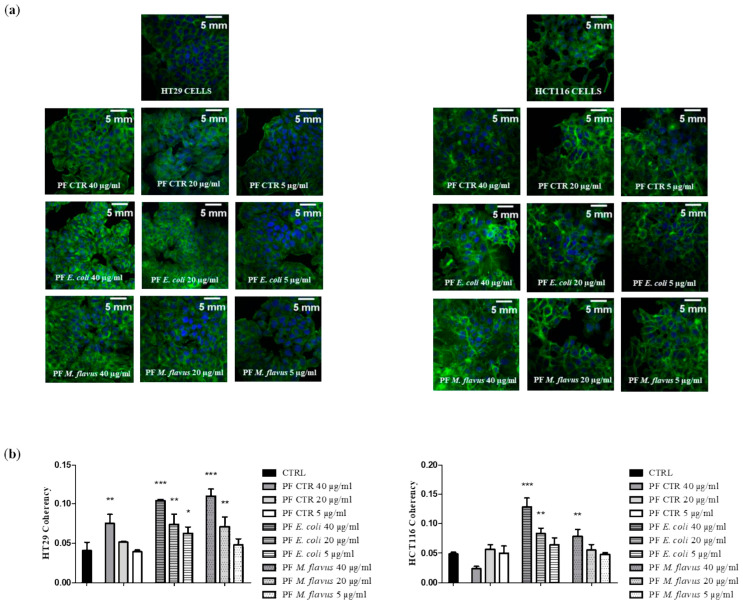
*H. illucens*-derived peptide fractions increased cytoskeleton coherency of human CRC cells. Cytoskeleton organization was affected by treatment with peptide fractions at different time points for both cell lines, as assessed by analyzing the actin fiber coherency in the control and treated cultures. Coherence was calculated from the tensor structure of each pixel in the image and is bounded by 0 (isotropic areas) and 1 (highly oriented structures), and the analysis was conducted on the original confocal images, according to the procedure described in the Materials and Methods Section. (**a**) Representative images of a cytoskeleton after 48 h of treatment: actin filaments are shown in green, while cell nuclei are stained in blue. Scale bars: 5 mm. (**b**) Data are presented as means ± the Standard Error (SE) of three replicates of three independent assays. Statistical significance between groups was evaluated by one-way analysis of variance (ANOVA), followed by Dunnett’s post hoc test (GraphPad Prism 5 software). The difference was considered statistically significant at * *p* < 0.05, ** *p* < 0.01, and *** *p* < 0.001.

To investigate whether the cytoskeleton organization was affected by treatment with peptide fractions, we analyzed actin fiber coherency in the control and treated cultures. Motile cells must assemble their cytoskeletal actin filaments in a spatially organized way, such that net filament growth and cell protrusion occur at the front of the cell. Coherency was calculated from the structure tensor of each pixel in the image and was bounded by 0 (isotropic areas) and 1 (highly oriented structures). We found that the peptide fractions induced an important reorganization of the actin filaments of cells’ cytoskeletons, with coherency increasing with increasing concentrations of the samples, and this effect was the highest with the peptide fractions obtained from infected larvae compared with lower concentrations of the samples and the control cells in both the HCT116 and HT29 cells (Figure 7).

### 2.4. Peptide Fractions Enhanced Cytotoxicity of Chemotherapeutic Drugs in Human CRC Cells

We aimed to evaluate whether hemolymph-derived peptide fractions could affect the cytotoxic activity of Oxaliplatin (OXA) and 5-Fluorouracil (5-FLUO), two well-known chemotherapeutic drugs that are widely used for treating CRC.

**Figure 8:**
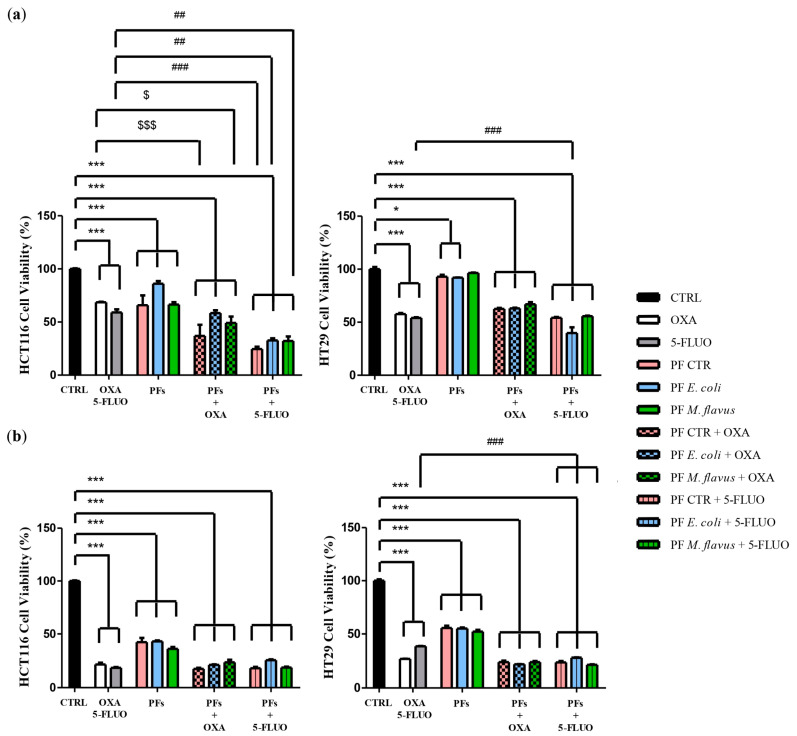
Effects of chemotherapeutic drugs and peptide fractions on HCT116 and HT29 cell viability. HCT116 (left) and HT29 (right) cells were exposed to Oxaliplatin (OXA) or 5-Fluorouracil (5-FU), alone or in combination with peptide fractions from larvae infected with *E. coli* or *M. flavus* and from uninfected larvae, and cell viability was assessed after (**a**) 48 h and (**b**) 72 h using the MTT assay. The percentage of viable cells was calculated as the ratio of treated cells to control cells. The data are expressed as means ± the Standard Error (SE) of three technical replicates of three independent assays, and statistical significance was evaluated (GraphPad Prism 5 software) with one-way ANOVA, followed by Dunnett’s post hoc test. Significance: * *p* < 0.05, and *** *p* < 0.001 for all samples vs. CTRL; ^$^ *p* < 0.05, and ^$$$^ *p* < 0.001 for all PF samples in combination with OXA vs. OXA; and, ^##^ *p* < 0.01, and ^###^ *p* < 0.001 for all PF samples in combination with 5-FU vs. 5-FU.

MTT assay was used to evaluate the growth inhibition of Oxaliplatin and 5-Fluorouracil, alone or in combination with peptide fractions, on the human CRC lines HT29 and HCT116. We compared the IC50 for Oxaliplatin and 5-Fluorouracil when used alone and the IC30 when used in combination with 20 µg/mL of peptide fractions obtained from infected or uninfected larvae. As shown in Figure 7, the combination with peptide fractions significantly increased the growth-inhibitory effect of both drugs in a dose- and time-dependent manner (Figure 8).

## 3. Discussion

The development of new antineoplastic drugs is a hot topic in cancer research and is crucial to override the limitations of current therapies, such as the development of resistance and toxicity [32].

Therapy for colorectal cancer cells (CRC), as well as other cancers, has evolved significantly over the years, with a range of treatment options available depending on the stage, location, and molecular characteristics of the tumor [33]. Oxaliplatin and 5-Fluorouracil (5-FU) are two chemotherapy drugs commonly used in the treatment of CRC patients, either alone or in combination with other agents. Although the development of targeted therapies, immunotherapies, and personalized treatment approaches has led to improved outcomes and survival rates for CRC patients, there is still room for improvement in terms of effectiveness and tolerability [34]. New approaches that are able to tackle both the toxicity issues of chemotherapy and resistance are of primary relevance for CRC [35].

In the past, the screening of natural products has allowed for the identification of some of the most active antitumor compounds (i.e., anthracyclines, Vinca alkaloids, epipodophyllotoxins, and taxanes). Thus, although current research in this area is more focused on the development of drugs specifically targeting molecular mechanisms involved in tumorigenesis, the screening of natural products might still represent a valid alternative. Moreover, natural products could represent a source of inspiration for the development of new antitumor drug candidates that are able to override resistance to currently available drugs and that are potentially characterized by increased selectivity on tumor cells and reduced side effects on healthy cells [36].

Insects represent a source of bioactive molecules, some of which have been used in traditional medicine for centuries and still provide a valuable supply of healing substances in less-developed countries [37,38,39,40,41,42,43,44,45,46,47,48]. The search for sustainable sources of biomolecules that find applications in cancer fields is currently a goal of increasing importance. The class of insects, which is distinguished by the abundance and variety of its molecules and activities, is one of the greatest sources of antimicrobial peptides (AMPs) [25]. Because they have an array of cellular and humoral responses from their innate immune system, insects are exceptionally well-adapted organisms to a wide variety of settings. Insects can also consume materials that vary in their degree of contamination; therefore, in order to combat these infections and endure hazardous environments, they produce AMPs [26]. Several insect-derived antimicrobial peptides (AMPs) have been reported in the literature and have also been suggested as promising anticancer peptides (ACPs) that potentially lack toxicity to healthy cells and that are unaffected by common mechanisms of resistance [49,50,51]. Studies have elucidated their mechanisms of action, which primarily involve targeting cancer cell membranes, disrupting their integrity, and inducing apoptosis or necrosis [13,26,52].

In this study, peptide fractions were obtained from the hemolymph of *Hermetia illucens* L. (Diptera: Stratiomyidae) larvae infected with strains of *Escherichia coli* or *Micrococcus flavus* and from uninfected larvae. Peptide fractions contain AMPs with a molecular weight below 30 kDa, which, according to numerous published studies in the literature, are the most active among the entire pool of higher-molecular-weight proteins in terms of both their antimicrobial effect [53,54] and their toxicity towards tumor cells [55,56].

Insect peptide fractions could represent a large reservoir of unexplored compounds with possible anticancer activity (ACPs). While the anticancer activity of natural ACPs isolated from other sources has been previously explored, less extensive is the literature specifically regarding the therapeutic potential of ACPs isolated from insects [8,57,58,59,60,61].

In our study, we evaluated and characterized the effects of peptide fractions from infected and from uninfected larvae of *H. illucens* on in vitro models of CRC cells. We previously characterized, by LC-MS/MS, the peptide fractions, identifying 33 AMPs: a total of 20 AMPs were expressed in all the analyzed conditions, 6 were expressed only after infection with *E. coli* or *M. flavus*, 1 was differentially expressed after infection with *E. coli*, and 6 were differentially expressed after infection with *M. flavus*. We identified defensins; cecropins; and attacins, a salivary-rich peptide and an uncharacterized protein (with antimicrobial activity).

Firstly, we observed that the peptide fractions strongly affected cell cycle progression, causing an accumulation of cells in the G2/M phase and showing a similar overall effect in both the cell lines tested. Furthermore, the peptide fractions induced an important reorganization of the cytoskeleton, leading to reduced motility. These effects were more evident with the peptide fractions obtained from the infected larvae compared to the uninfected ones and reached the highest significance with the peptide fractions obtained from the larvae infected with *E. coli*.

Similar effects were observed in both the CRC cell lines tested. However, apoptosis was induced in the HCT116 but not in the HT29 cancer cells, thus suggesting that the effects of peptide fractions can be affected by the cellular context. It is noteworthy that HCT116 is a highly aggressive cell line with a reduced capacity to differentiate compared to HT29 cells [62], and, unlike HT29, it carries a KRAS mutation, which is a frequent and early event in CRC tumorigenesis [63]. In addition, it could be possible that a p53 mutation can also influence the response of CRC cells to the induction or non-induction of apoptosis. Indeed, HCT116 is a wild-type p53 colon-cancer cell line (with a functional p53 protein), whereas the HT29 cell line carries a p53 mutation, which may lose its ability to trigger apoptosis in response to cellular stresses, such as treatment with chemotherapeutic drugs or other pro-apoptotic substances [64,65]. Several insect-derived AMPs have been reported to induce apoptosis in different tumor cells. Jin et al. [66] showed that cecropin, derived from *Musca domestica*, was able to induce apoptosis and inhibit the proliferation of human hepatocellular carcinoma BEL-7402 cells in dose-dependent and time-dependent manners, without affecting the proliferation of normal liver cells [67]. CopA3, a defensin-like antimicrobial peptide identified from the beetle *Copris tripartitus*, was shown to induce the apoptosis of different cancer cells via a caspase-independent pathway [67,68]. The anticancer activity of ACPs towards CRC cells is probably exerted by different cell biochemical pathways giving rise to multiple biological effects. In the literature, in addition to apoptosis, other mechanisms of action of ACPs have been reported. Two peptides isolated from *Calliphora vicina* showed immunomodulatory effects in vivo on mice infected with P388 murine leukemia cells, stimulating natural killer cells and IFN synthesis [24]. However, still little is known about the molecular effects of insect protein extracts on cancer cells, and the identification and purification of specific ACPs from peptide fractions could lead to stronger and more suitable anticancer effects. The observed effects on cell motility and cytoskeleton coherence are equally interesting, as both features play an important role in the migration and invasion of cancer cells into adjacent and distant tissues in the metastatic process. We analyzed AMP effects on in vitro models of purified CRC cell lines; therefore, we could not specifically assess their potential immunomodulatory effects, such as macrophages or T cell activation within the tumor microenvironment. However, it is noteworthy that numerous studies have demonstrated that AMPs can modulate immune responses by acting as damage-associated molecular patterns (DAMPs), promoting the activation of innate immune cells, including macrophages and dendritic cells [69,70,71,72,73]. Thus, future studies are warranted to evaluate whether AMPs derived from *H. illucens* may exert immunomodulatory effects which would contribute to their potential anticancer effects in vivo, in addition to their direct cytotoxic effects on cancer cells.

Finally, combination studies have demonstrated that protein fractions can enhance the cytotoxic effects of 5-Fluorouracil and Oxaliplatin. Our results suggest that a combination treatment with insect-derived protein fractions warrants further studies, since this might represent a potential therapeutic strategy for CRC by ensuring better efficacy on cancer cells at lower doses of chemotherapeutics and, therefore, with a lower risk of side effects and toxicity. These effects may be even better if the specific components of the fraction, previously identified, can be isolated and tested singularly or in combination. These results are of great interest, since both drugs are widely used in the treatment of CRC as well as other cancers, but their application is often restricted due to toxic side effects and/or drug resistance in clinical practice [74]. We did not observe the occurrence of resistance in CRC cells following repeated exposure to the peptide fractions from *H. illucens*. This may be due to the short duration of the performed experiments, and further studies are warranted to evaluate whether CRC cells may develop resistance to AMPs following prolonged exposure. However, it is noteworthy that one of the key advantages of AMPs is their reported low propensity to induce resistance compared to conventional chemotherapeutics, since they act through fundamentally different mechanisms [75,76,77,78,79,80,81,82,83,84,85,86]. Indeed, unlike many traditional anticancer agents that require internalization to reach intracellular targets, AMPs exert their effects extracellularly, primarily targeting cancer cell membranes and inducing rapid cell lysis [87,88,89,90,91]. This short interaction time and the direct disruption of the plasma membrane significantly reduce the likelihood for cancer cells to develop adaptive resistance, a common phenomenon with conventional therapies. Additionally, AMPs can bypass key resistance mechanisms, such as multidrug-resistant (MDR) efflux pumps, since their cytotoxicity is largely independent of intracellular drug accumulation. All these aspects further support the hypothesis that AMPs could display useful advantages over conventional anticancer therapies and support the great interest in the field that is aimed at AMPs’ characterization and at assessing their safety, efficacy, and clinical benefits alone and in combination therapies [71]. In conclusion, our findings lay the foundation for undertaking new studies to identify specific AMPs that are present in insect-derived hemolymphs and to extend these studies to other components of the inexhaustible source of the world of insects for the benefit of cancer patients.

## 4. Materials and Methods

### 4.1. Infection of H. illucens Larvae, Hemolymph Extraction, and Precipitation by Organic Solvents

The larvae of *Hermetia illucens* (Diptera: Stratiomyidae), provided by Xflies s.r.l (Potenza, Italy), were washed with distilled water and 70% ethanol and, subsequently, to stimulate production of antimicrobial peptides (AMPs), were infected by injection into the hemocelic cavity, with fine glass capillaries immersed in a suspension of the Gram-positive bacteria *Micrococcus flavus* (DSM 1790; cultured to exponential phase OD600 = 1) or of the Gram-negative bacteria *Escherichia coli* (LGM 2092; cultured to exponential phase OD600 = 1) and were incubated at 25 °C and 70% of relative humidity for 24 h. Uninfected larvae were considered controls. After the incubation period, the hemolymph of the infected and uninfected larvae (used as controls) was extracted in tubes containing *L*-ascorbic acid to prevent the melanization process of the hemolymph and was kept on ice until the end of the procedure. The tubes were then centrifuged at 10,000 rpm for 5 min at 4 °C to recover the plasma and remove the cellular component and any debris.

The protein component in the hemolymph was precipitated by organic solvents, including methanol, acetic acid, and distilled water, as reported in Scieuzo et al. [30], obtaining a supernatant containing peptides with a molecular weight lower than 30 kDa and a pellet with proteins of a higher molecular weight. The supernatant was collected in new tubes, vacuum-dried to remove the organic solvents, and resuspended in a volume of sterile water that was equal to the original plasma volume. All samples thus prepared, containing the peptide fractions (PFs) derived from hemolymphs extracted, respectively, from uninfected larvae (PF CTR) and larvae infected with *E. coli* (PF *E. coli*) and *M. flavus* (PF *M. flavus*), were stored at −20 °C until their subsequent use [30].

The amount of peptides was evaluated by the Bradford assay, and the concentration was standardized at 2 µg/µL for all samples [92].

### 4.2. Cell Culture

HT29 (RRID:CVCL_0320) and HCT116 (RRID:CVCL_0291) human colorectal adenocarcinoma and carcinoma (CRC) cell lines were obtained from American Type Culture Collection (ATCC, Manassas, VA, USA) and were routinely tested for mycoplasma. They were cultured in DMEM (Dulbecco’s Modified Eagle’s Medium, Euroclone, Pero, MI, Italy), supplemented with 10% Fetal Bovine Serum (FBS; GIBCO (Thermo Fisher Scientific, Waltham, MA, USA)); 1% penicillin-streptomycin (Euroclone); and 2 mmol/L of *L*-glutamine (Euroclone, Pero, MI, Italy) at 37 °C in a humid, 5% CO_2_ atmosphere.

### 4.3. Measurement of Cell Viability

Cell viability was evaluated by using the MTT [3-(4,5-dimethylthiazol2-yl)-2,5-diphenyltetrazolium bromide], Merk Life Science (Sigma-Aldrich, St. Louis, MO, USA), colorimetric assay. Cells were seeded in 96-well microtiter plates (8 × 10^3^ cells per well) and were incubated for 24 h. The medium was then replaced to treat the cells for 48 h and 72 h with decreased concentrations (40 µg/mL, 20 µg/mL, 10 µg/mL, 5 µg/mL, 2.5 µg/mL, 1.25 µg/mL, 0.62 µg/mL, and 0.31 µg/mL) of peptide fractions obtained from larvae infected with *E. coli* or with *M. flavus* and from uninfected larvae. The concentrations of 40 µg/mL, 20 µg/mL, and 5 µg/mL were used to assess all subsequent analyses.

An MTT solution was added to each well and incubated at 37 °C for 2 h. The total amount of MTT formazan crystals was measured using a microplate reader (SpectraMax Plus 384 spectrophotometer (Molecular Devices Corp., Sunnyvale, CA, USA)) to measure absorbance at 570 nm and at a reference wavelength of 630 nm. The crystals were then dissolved by adding a stop solution of HCl and isopropanol. By the GraphPad Prism software, the 50% inhibitory concentration (IC50) was determined.

Oxaliplatin (OXA) and 5-Fluorouracil (5-FU), at the IC30 concentration of 8 µM and 48 µM, respectively, were used to evaluate the possible synergistic effect of peptide fractions with chemotherapy drugs. As a negative control, a fresh medium was added in the control wells.

### 4.4. Western Blot Analysis

Treated cells were lysed using a lysis buffer (Cell Signaling Technology, Danvers, MA, USA) (50 mmol/L of Tris-HCl; pH 7.2; 5 mmol/L of MgCl_2_; 50 mmol/L of NaCl, 0.25%; 0.1% SDS; and 1% Triton X-100) complemented with protease inhibitors (2 mmol/L of phenyl methyl sulfonyl fluoride (PMSF), 10 mg/mL of aprotinin, 2 mg/mL of leupeptin, 2 mmol/L of Na_3_VO_4_, and 100 mmol/L of NaF). Using the Bradford technique (Bradford Protein Assay Kit II, BioRad, Hercules, CA, USA) and bovine serum albumin (BSA) as a standard, the amount of protein was measured, and the same amounts of cell lysates (30 µg) were then transferred to PVDF (GE Healthcare, Solingen, Germany) blotting membranes after being resolved by SDS-PAGE (Sodium Dodecyl Sulfate Polyacrylamide Gel Electrophoresis). The membranes were probed with the primary antibodies, were incubated overnight, and were analyzed using the enhanced chemiluminescence kit for Western blotting detection (Advansta, WesternBright TM ECL, Bering Drive San Jose, CA, USA). Primary monoclonal antibodies were used following the suppliers’ instructions and included the following: mouse anti-human monoclonal GAPDH (H-284; sc-30220; dilution, 1:500; Santa Cruz Biotechnology, Inc., Dallas, TX, USA); mouse monoclonal anti-human β-Actin (C-4; sc-47778; dilution, 1:500; Santa Cruz Biotechnology, Inc.); cleaved Caspase-3 (Asp175) (5A1E) Rabbit mAb (9664; dilution, 1:1000; Cell Signaling Technology, Danvers, MA, USA); mouse monoclonal anti-human PARP-1 (N-20; sc-1561; dilution, 1:500; Santa Cruz Biotechnology, Inc.); monoclonal anti-human p21 (M-19; sc-471; dilution, 1:500; Santa Cruz Biotechnology, Inc.); and mouse monoclonal anti-human cyclin D1 (72-13G; sc-450; dilution, 1:200; Santa Cruz Biotechnology, Inc.).

### 4.5. Alkaline Phosphatase (AP) Assay

Alkaline phosphatase level (APL) activity was evaluated in the CRC cells using a Phosphatase Assay Kit (G-Biosciences, Inc., St. Louis, MO, USA), according to the manufacturer’s instructions. This kit uses para-nitrophenyl phosphate (pNPP) as a phosphatase substrate, which turns yellow (λmax = 405 nm) when dephosphorylated by ALP.

Experimentally, cells were treated for 48 h with different concentrations (40 µg/mL, 20 µg/mL, and 5 µg/mL) of peptide fractions obtained from larvae infected with *E. coli* or *M. flavus* and from uninfected larvae (PF CTR), before being lysed in ice with 1% Triton X-100 for 20 min. Then, 50 µL of the total cellular lysates were incubated for 30 min at room temperature with 50 µL of an AP substrate and 50 µL of an AP assay buffer, and, at the end of the incubation, the reaction was stopped, with 50 µL of a stop solution, and the OD values were read at 405 nm for 1 h. Spectrophotometric readings were taken on a SpectraMax Plus 384 spectrophotometer (Molecular Devices Corp., Sunnyvale, CA, USA) and were analyzed using the Softmax Pro Software version 4.3 (Molecular Devices Corp.) to obtain the Vmax values. The relative AP activity was normalized by the total protein concentration.

### 4.6. Apoptosis and Cell Cycle Analysis

HT29 and HCT116 cells were seeded on 60 mm plates (5 × 10^5^ cells per plate) and were incubated for 24 h. Afterwards, they were treated with different concentrations (40 µg/mL, 20 µg/mL, and 5 µg/mL) of peptide fractions obtained from larvae infected with *E. coli* or *M. flavus* and from uninfected larvae for 48 h. Untreated cells represented negative controls.

For the apoptosis assay, the cells were washed once with cold PBS, were harvested, were collected in new tubes in ice, and analyzed using the Dead Cell Apoptosis Kit with Annexin V FITC and Propidium Iodide for flow cytometry (Invitrogen, Thermo Fisher Scientific, Waltham, MA, USA), according to the manufacturer’s instructions. Analyses were carried out using a CytoFLEX benchtop flow cytometer (Beckman Coulter, Cassina de’Pecchi, Milano, Italy), and data were analyzed with the Kaluza 2.1 analysis program.

For a cell cycle analysis, cells were washed once with cold PBS, were harvested, were collected in new tubes in ice, were filtered, and were resuspended in 80% ethanol drop by drop. After centrifugation at 1300 rpm for 5 min at 4 °C, the pellets were washed with cold PBS without calcium and without magnesium and were analyzed using the Cell Cycle Kit (Beckman Coulter), according to the manufacturer’s instructions. Analyses were carried out using a Cytoflex flow cytometer (Beckman Coulter). Data were analyzed with the Kaluza 2.1 analysis program.

### 4.7. Confocal Microscopy

HCT116 and HT29 cells were grown in 24-well plates (5 × 10^4^ cells/well) on glass coverslips that were previously washed with absolute ethanol for 24 h. After a period of incubation, the cells were treated with peptide fractions, and untreated cells were used as a negative control. After 48 h of treatment, the cells were washed twice with cold PBS and were fixed with 4% paraformaldehyde for 30 min. The coverslips, after permeabilization with triton X-100 0.1%/PBS for 5 min, were blocked with a 3% normal goat serum/PBS for 30 min at room temperature. Then, to counterstain, cytoskeleton coverslips were incubated with fluorescein isothiocyanate-labeled phalloidin at room temperature for 45 min and in the dark. Lastly, the slides were assembled with DAPI (Fluoromount G with DAPI; Electron Microscopy Sciences, Hatfield, PA, USA). Slides were examined using an inverted confocal microscope (Nikon Eclipse Ti2, Nikon, Tokyo, Japan) equipped with an environmental control system (Okolab, Naples, Italy). Images were taken using the ImageJ software version 1.53t (NIH, Bethesda, Rockville, MD, USA). Analyses of coherency were performed using the OrientationJ plugin for ImageJ/Fiji software.

### 4.8. Wound-Healing Assay

Cells were seeded (2 × 10^5^ cells per well) in 24-well plates and were allowed to attain confluence before a manual scratch assay. A scratch wound was produced manually with a pipette tip, and the media were changed with media containing peptide fractions obtained from larvae infected with *E. coli* or *M. flavus* and from uninfected larvae and fresh media for negative controls. Wounds were photographed (Nikon Eclipse TE2000-U) at five time points (0 h, 6 h, 12 h, 24 h, and 48 h).

### 4.9. Statistical Analysis

Data are shown as means ± the Standard Error (SE) of three technical replicates of three independent assays. Statistical significance between groups was assessed by a one-way analysis of variance (ANOVA), followed by Dunnett’s post hoc test by the GraphPad Prism 5 software. The difference was considered statistically significant at * *p* < 0.05, ** *p* < 0.01, *** *p* < 0.001, and **** *p* < 0.0001; ^#^ *p* < 0.05, ^##^ *p* < 0.01, ^###^ *p* < 0.001; ^$^ *p* < 0.05, ^$$^ *p* < 0.01, ^$$$^ *p* < 0.001.

## Data Availability

The data sets used and/or analyzed for the current study are available from the corresponding author on reasonable request.

## Supplementary Materials

The following supporting information can be downloaded at: https://www.mdpi.com/article/10.3390/ijms26051891/s1.
