# Supplementary material for: Peptide Fractions Extracted from the Hemolymph of Hermetia illucens Inhibit Growth and Motility and Enhance the Effects of Traditional Chemotherapeutics in Human Colorectal Cancer Cells
Source: Int J Mol Sci. 2025 Feb 22;26(5):1891. doi: 10.3390/ijms26051891 (PMC11899838; doi:10.3390/ijms26051891)
Supplement: Supplementary file 1 [file ijms-26-01891-s001.zip › ijms-3454429-supplementary.pdf]

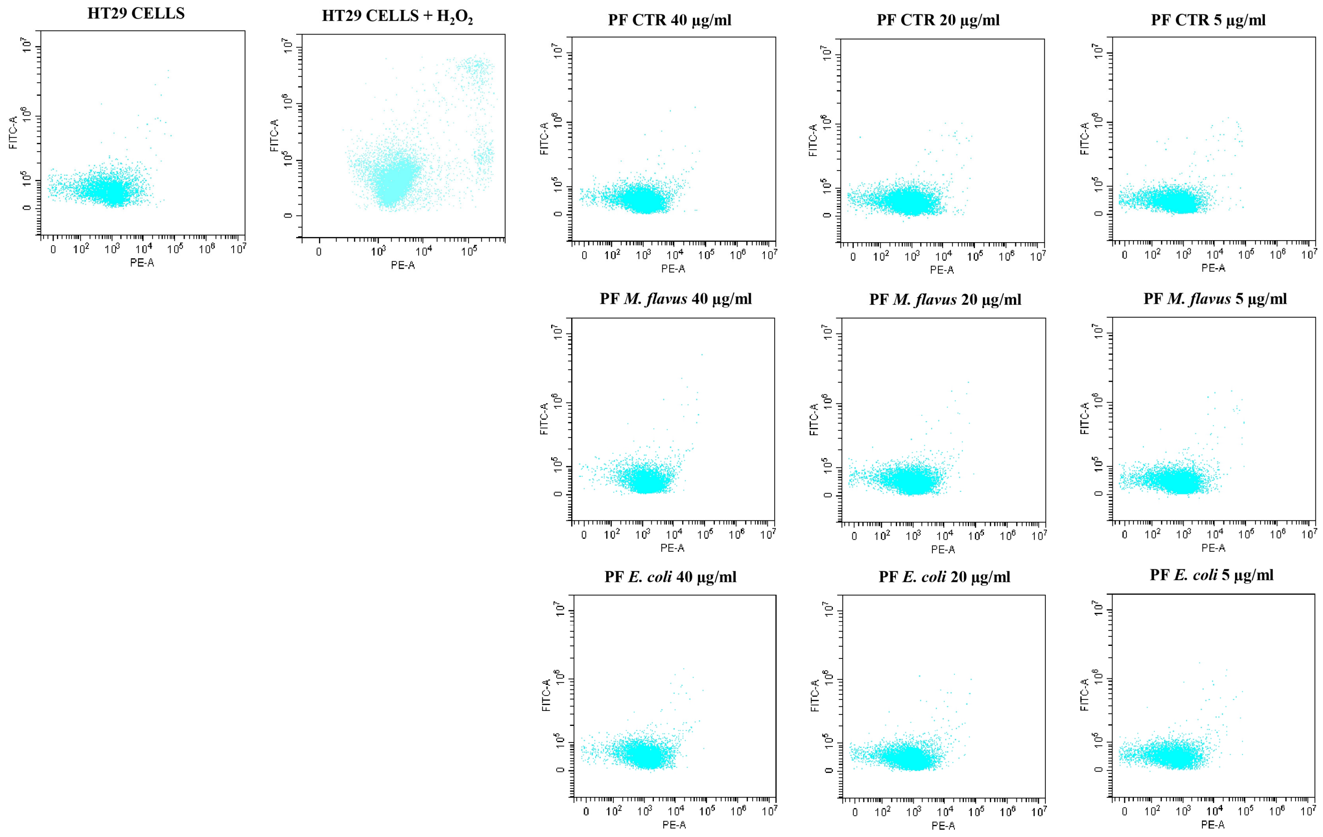

Supplementary Figure S1: Flow cytometry analysis of apoptosis in HT29 cells treated with peptide fractions. HT29 cells were treated for 48 h with the indicated increasing concentrations of 40, 20, and 5 µg/ml of peptide fractions obtained from *Hermetia illucens* larvae infected with *Escherichia coli* (PF *E. coli*), *Micrococcus flavus* (PF *M. flavus*), or from uninfected larvae (PF CTR). Representative dot plots show no induction of apoptosis in HT29 cells upon treatment.
